# Vaccination in Older Adults: The When and Where

**DOI:** 10.1093/geroni/igaa057.2928

**Published:** 2020-12-16

**Authors:** Sara Poston

**Affiliations:** GSK Vaccines, Philadelphia, Pennsylvania, United States

## Abstract

Despite the well-understood benefits of vaccination in older adults, national rates still fall below public health targets, especially among certain racial and ethnic groups. Recent scholarship examining healthcare use patterns in adults revealed that health care providers miss several opportunities to provide vaccination during regular healthcare encounters, including Medicare annual wellness visits. Several barriers to older adult vaccination have been identified, including lack of patient and provider understanding of the importance of vaccination, financial barriers to vaccines covered under Medicare Part D, and patient hesitancy about the safety and effectiveness of vaccines. Strategies to address these barriers will be discussed, including the use of national quality measures to strengthen incentives for adult vaccination. Part of a symposium sponsored by the Health Behavior Change Interest Group.

